# Characterizing menstrual health and education needs among menstruating adolescents: A cross-sectional study on dysmenorrhea, cycle irregularities, and menstrual education

**DOI:** 10.1016/j.dialog.2026.100327

**Published:** 2026-07-27

**Authors:** Idhaliz Flores-Caldera, Kathleen N. Morales, Braulio I. Rivera, Maricarmen Colón-Díaz, Yeidelin Nieves, Christy Rosado, Maria F. Martínez, Adriana Diaz-Betancourt, Angela Cooke-Jackson, Valerie Rubinsky

**Affiliations:** a Department of Basic Sciences, Ponce Health Sciences University, Ponce, PR 00732, USA; b Department of ObGyn, Ponce Health Sciences University, Ponce, PR 00732, USA; c Department of Public Health, Ponce Health Sciences University, Ponce, PR 00732, USA; d Department of Physiology/Pathology, San Juan Bautista Medical School, Caguas, PR 00726, USA; e University of Puerto Rico, Ponce Campus, Ponce, PR 00732, USA; f Brain and Behavioral Sciences, Ponce Health Sciences University, Ponce, PR 00732, USA; g Department of Public Health, California State University, Los Angeles, CA 90032, USA; h Speech Department, Community College of Allegheny County, Augusta, ME 04330, USA

**Keywords:** Menstrual health, Menstrual cycle, Menstruation, Menstrual education, Female adolescents

## Abstract

**Purpose:**

Recognizing normal and abnormal menstrual features is essential for the early detection of gynecologic conditions, facilitating timely medical care and preventing associated biopsychosocial impacts. However, there is limited information on menstrual cycle characteristics, clinical profiles, and education among menstruating adolescents in Puerto Rico, as well as how these factors relate to sociodemographic factors such as age, school type (public/private), region of residence (metropolitan/non-metropolitan and residential zone (urban/rural).

**Methods:**

This cross-sectional study was designed to assess the menstrual cycle characteristics, symptoms and dysregulations, self-reported menstrual conditions, and sources of menstrual education available to menstruating adolescents across Puerto Rico. An anonymous online survey designed by the study team was disseminated via social media, text messaging, and in collaboration with the Department of Education of Puerto Rico. Differences in the likelihood of receiving education, sources, trusted figures, and level of understanding and comfort regarding menstrual health topics and clinical outcomes (moderate-severe menstrual pain, emergency room (ER) utilization) based on sociodemographics factors were assessed by logistic regressions.

**Results:**

Analysis of data from 1094 menstruating adolescents showed high rates of menstrual symptoms, particularly severe dysmenorrhea and cycle irregularities, with substantial impacts on daily life. Participants living in urban areas had higher odds of reporting prior menstrual health education. Older participants were more likely to receive information through school classes, social media, and physicians. Students in public schools had lower odds of reporting media and physicians as key sources or trusted contacts. Comfort discussing menstrual health topics increased with age, while perceived understanding of the menstrual cycle did not differ significantly across sociodemographic groups. Moderate to severe menstrual pain was not associated with sociodemographic factors, whereas ER visits varied by age, region, and school type that may determine access to care.

**Conclusion:**

This study revealed high prevalence of menstrual irregularities and pain among menstruating adolescents, emphasizing the need for improved menstrual health education and implementation of early intervention strategies. Significant disparities in education, trusted figures, comfort and ER utilization across demographics demonstrate the importance of tailoring menstrual health initiatives to address the unique needs of this population.

## Highlights

• Severe dysmenorrhea and cycle irregularities were highly prevalent, revealing a substantial, underrecognized health burden.
• Menstrual education, trust, and comfort discusing it varied by age, school and location--interventions must be targeted.
• Pain severity did not differ by group, but ER use varied by age, region, and school type, revealing care access inequities.

## 1 Introduction

The menstrual cycle is a natural biological process that women and individuals with a uterus experience over several decades of their reproductive lives. Understanding both the normal and abnormal features of the menstrual cycle is crucial for timely diagnosis, prompt medical care, and the prevention of negative biopsychosocial impacts. During adolescence, disruptions in the menstrual cycle—irregularity, heavy bleeding, and dysmenorrhea (defined as pelvic pain during menses)—are common and often resolve with time. However, medical intervention is often necessary [1]. For instance, up to 70% of female adolescents with dysmenorrhea unresponsive to first-line treatments [e.g, oral contraception (OCP)], may have endometriosis, a debilitating condition that can severely impact daily life if left untreated [2], [3]. Delays in diagnosis and treatment of conditions such as endometriosis and polycystic ovarian syndrome (PCOS), which substantially impact the daily lives of female adolescents, underscore the importance of early diagnosis and treatment, which are critical for optimal menstrual health defined as “a state of complete physical, mental, and social well-being in relation to the menstrual cycle” [4].

Despite the critical need for menstrual health education as a tool for early detection and intervention of gynecologic disorders, access to comprehensive and adequate information is often insufficient, especially during adolescence, when menstrual periods significantly impact daily life [5]. Research indicates that while 70% of patients diagnosed with endometriosis report experiencing pain before the age of 20, and ∼ 40% before the age of 15, the average time to diagnosis remains between 5 and 10 years. This delay is likely influenced by the dismissal or normalization of painful and disruptive menstrual symptoms, as well as systemic factors such as deficits in menstrual education and faulty referral pathways to expert care [6], [7]. Cultural norms and media often perpetuate the idea that menstrual cramps are an inevitable part of the menstrual cycle, making it difficult for young people and their caregivers to distinguish between normal discomfort and symptoms that warrant medical attention [8], [9]. Mothers are the primary source of menstrual education for adolescent females, even though the information they provide might be insufficient or inaccurate, often leading to the normalization of menstrual dysregulations based on their own personal experiences with menstruation [10]. Even when menstrual health education is provided in schools, it often fails to address the variability of menstrual experiences and their emotional impact [11].

With the increasing use of social media, adolescents are frequently exposed to both accurate and misleading information about menstruation and menstrual health [9], [12]. Worryingly, those who receive medically inaccurate information are more likely to develop negative expectations about menarche and menstruation, leading to lasting implications on their health and well-being [13]. Studies have shown that improving menstrual health literacy can destigmatize this topic, reduce menstruation-related stress, and increase self-efficacy [14], [15]. To bridge educational gaps, a comprehensive curriculum is essential, offering practical information and guidance on managing menstrual disorders and related symptoms. This curriculum should be delivered in a safe, supportive environment that encourages questions and provides resources to empower effective menstrual health management [11], [16]. Furthermore, menstrual health education must extend beyond female students to include male students, teachers, and caregivers, ensuring a holistic and inclusive approach to improving menstrual health literacy [17].

There are no data on the characteristics of the menstrual cycle or the prevalence of menstrual symptoms, cycle dysregulations, and gynecologic conditions among menstruating adolescents in Puerto Rico, a commonwealth of the US that is part of the Higher Antilles in the Caribbean region. This study was designed to fill this gap, while also assessing the primary sources of menstrual health information, trusted figures, and perceived level of understanding and comfort when discussing menstrual health topics. Also, our study assessed factors associated with having received menstrual health education; and feeling comfortable discussing menstruation and related topics with others; reporting moderate to severe menstrual pain; and visiting an emergency department. Our study provides critical insights on current gaps in menstrual health profiles and education that will guide future development of tailored educational programs to improve early identification, management, and outcomes of menstrual disorders in adolescent populations.

## 2 Methods

### 2.1 Ethics

The study protocol, survey and recruitment materials were approved by the Institutional Review Board (IRB) Committee of the host institution (IRB approval # 2405199132). Participants read an assent statement outlining the study purpose, risks, and voluntary nature prior to answering an age-appropriate anonymous electronic survey. In accordance with federal regulations for minimal-risk research involving adolescents (45 CR 46.408c), the IRB approved a waiver of parental permission given that 1) the survey did not collect information that could identify participants, 2) there were no sensitive questions, 3) except age (which was inclusion criteria), questions were not required and could be skipped and the survey closed at any time, ensuring minimal risks associated with voluntary completion of the survey. Those assenting to participate checked the “submit” button taking them to the online survey. No incentives were provided for participation. Privacy safeguards included the anonymous REDCap survey that did not involve device capture, secure data storage in the Ponce Research Institute REDCap network, protected by firewalls and passwords, as well as restricted access only to key study personnel.

### 2.2 Study design

This cross-sectional study involved distributing an electronic link to the anonymous study questionnaire via social media (Instagram and Facebook of the PRI), and text messaging (WhatsApp) using a snowball non-probabilistic recruitment strategy. Permissions were obtained from the Department of Education of Puerto Rico to disseminate the study's promotional flyer during the Health & Development class for Middle and High School students. The survey was open from January 2023 to April 2024. We followed the STROBE guidelines to ensure adherence to quality standards of cross-sectional studies.

### 2.3 Study participants

Recruitment materials targeted Puerto Rican adolescents 11 to 20 years old (minors under the PR law) who menstruate using feminine language in Spanish. Because sex assigned at birth and gender identity were not assessed separately, the study cannot distinguish between these constructs.They must own a smartphone or mobile device to participate as the digital survey was disseminated using a QR code.

### 2.4 Survey development

The scientific team with expertise in women's health translational research and menstrual health education developed an anonymous electronic survey in REDCap to securely gather self-reported information about the menstrual cycle and menstrual education among our target population. The survey had four modules: (i) demographics, (ii) menstrual cycle characteristics, (iii) clinical profiles, and (iv) menstrual health education. Demographic questions included: age, type of school (public vs. private), region of residence (metropolitan vs non-metropolitan region) and residential zone (urban vs. rural). Menstrual characteristic questions included: (a) irregular cycles (“How often do you have your period on average? Write ‘irregular’ if it is too irregular to say exactly”; (b) chronic pelvic pain (CPP, “Have you experienced pelvic pain for more than 6 menstrual cycles in a row?”; (c) heavy/abundant flow (“Describe the amount of bleeding you usually experience during your period on days when it's heaviest” [a figure with different levels of flow was included for reference], and level of pain during menses (Numerical Rating Scale (NRS): *0* = no pain, 10 = the worst pain you can imagine). Clinical profile questions included: menstrual-related symptoms, gynecologic conditions diagnosed by a physician, treatments used for menstrual symptoms, impact of symptoms on daily life activities, and visits to Emergency Room (ER). Menstrual health education questions included: sources (both structured and informal), trusted figures, and self-reported level of understanding and comfort when discussing related topics using Likert scales.

Prior to distribution, the questionnaire was pilot tested with six adolescents to ensure clarity, comprehension, and age-appropriateness. The full survey in English and Spanish is available in Supplemental Materials.

### 2.5 Statistical analysis

Aim 1: Understand sociodemographics, menstrual cycle characteristics, menstrual clinical profiles of the overall sample. Data were analyzed using descriptive statistics, including frequencies and percentages for categorical variables and means with standard deviations (SD) for continuous variables. Corresponding 95% confidence intervals (95% CI) were calculated for key estimates, including proportions and mean values, to provide measures of precision.

Aim 2: Determine if menstrual health education features differ by sociodemographic factors (age, school type, region, zone). We hypothesized that sociodemographic variables are significantly associated with educational outcomes related to menstrual health. Age was entered into the logistic regression models as an ordinal variable based on ordered age groups. Region (metropolitan/non-metropolitan), residential zone (urban/rural), and school type (public/private) were entered as binary categorical variables. Logistic regression models were conducted to examine adjusted associations between sociodemographic factors and outcomes of interest: [1] having received menstrual education, [2] sources of menstrual education, [3] perceived understanding, [4] trusted figures, and [5] level of comfort discussing these topics with others.

Aim 3: Determine if clinical outcomes differ by sociodemographic factors (age, school type, region, zone). We hypothesized that sociodemographic variables are significantly associated with clinical outcomes related to menstrual health. Logistic regression models were conducted to examine adjusted associations between sociodemographic factors and outcomes of interest: [1] reporting moderate to severe menstrual pain; and [2] having visited an ER due to pelvic pain or menstrual-related symptoms. Covariates included in the models were selected a priori based on theoretical relevance and prior literature. Multicollinearity was assessed using variance inflation factors (VIF), and no evidence of problematic collinearity was observed. Model fit and calibration were evaluated using the Hosmer–Lemeshow goodness-of-fit test. Results from regression analyses are reported as odds ratios (OR) with 95% confidence intervals (CI).

For comparative analyses, effect sizes with 95% confidence intervals were reported where applicable (e.g., mean differences for continuous variables and odds ratios for categorical outcomes) to complement p–values and facilitate interpretation of the magnitude of associations. All statistical analyses were conducted using SPSS version 29.0; graphical representations were generated using GraphPad Prism version 10.3. Statistical significance was defined as *p* < 0.05.

## 3 Results

### 3.1 Data missingness

The survey shows a global missing answer rate of 12%. For items administered to the full sample, percentages were calculated using the total analytic sample (*N* = 1094) as the denominator, with skipped responses reported as a separate “Skipped” category. For items administered using branching logic (e.g., follow-up questions on pain severity, emergency department visits, treatment use) the denominator was the number of participants who met the branching criteria. All percentages for branched items therefore use the eligible subgroup as the denominator and the proportion of those who skipped the question is reported.

Missing data were handled using multiple imputation (*n* = 5 imputations) with fully conditional specification. Analyses were conducted using pooled estimates across imputed datasets. Sensitivity analyses comparing imputed and complete-case results showed consistent findings.

1) *Sociodemographics, menstrual cycle characteristics, menstrual cycle clinical profiles, and menstrual health education features of overall sample*:

*Sociodemographics.* A total of 1102 individuals accessed the survey link, and 1094 completed it. Participants were aged 11–20 years with a mean age of 15.7 years (SD ± 2.1; 95% CI: 15.5–15.8) and ranged from 6th grade to the 4th year of college. All seven educational regions of Puerto Rico were represented. Most participants (70.6%, 95% CI: 67.8%–73.3%) attended public schools and lived in urban areas (74.1%, [95%; 1.4%–76.7%) outside the metropolitan region of San Juan (60.6%, 95% CI: 57.6%–63.5%) (Puerto Rico Capital city) (Table 1).

**Table 1:** Demographic and menstrual cycle profile of study participants.

Variable	N = 1094	% (valid percent)	SD [95% CI]
Age groups (years old)			
Mean			2.1 [15.5–15.8]
11–14	332	30.4	[27.6%–33.2%]
15–17	597	54.6	[51.6%–57.6%]
18–20	156	14.3	[12.2%–16.5%]
Skipped	13	1.2	
Educational region			
Ponce	292	26.7	[24.1%–29.4%]
San Juan	237	21.7	[19.3%–24.2%]
Mayagüez	234	21.5	[19.0%–23.9%]
Bayamón	129	11.8	[9.9%–13.9%]
Humacao	42	3.8	[2.8%–5.2%]
Caguas	74	6.8	[5.4%–8.4%]
Arecibo	19	1.7	[1.1%–2.7%]
Skipped	66	6.0	
School type			
Public	772	70.6	[67.8%–73.3%]
Private	299	27.3	[24.7%–30.1%]
Other (homeschool, GED)	10	0.9	[0.4%–1.7%]
Skipped	13	1.2	
Residential zone			
Urban	811	74.1	[71.4%–76.7%]
Rural	257	23.5	[21.0%–26.1%]
Skipped	26	2.4	
Region			
Non-Metro	663	60.6	[57.6%–63.5%]
Metro	365	33.4	[30.6%–36.3%]
Skipped	66	6.0	
**Experienced menarche**	1065	97.4	[96.2%–98.2%]
Not yet experienced menarche	20	1.8	[1.1%–2.8%]
Skipped	9	0.8	
Mean age at menarche	11.9		1.7 [11.8–12.0]
Early menarche (8–10 yrs)	202	18.8	[16.7%–21.4%]
Late menarche (15–17 yrs)	102	9.5	[7.9%–11.5%]
Cycle regularity (*n* = 1074)			
Irregular Cycles	444	41.3	[38.4%–44.4%]
Regular Cycles	579	53.9	[50.9%–56.9%]
Not sure	48	4.5	[3.3%–5.9%]
Skipped	3	0.3	
Menstrual flow (n = 1074)			
Light	100	9.3	[7.6%–11.2%]
Moderate	555	51.7	[48.6%–54.7%]
Abundant/Profuse	412	38.4	[35.4%–41.4%]
Skipped	7	0.6	
**Period pain, Y (n = 1074)**	922	85.9	[83.6%–87.9%]
Severity level (*n* = 922)			
Severe	336	36.4	[33.3%–39.6%]
Moderate	408	44.3	[41.0%–47.5%]
Mild	163	17.7	[15.3%–20.3%]
Skipped	15	1.6	
Last menstrual period pain level, numerical rating scale (n = 922)			
0 = No pain	19	2.1	[1.2%–3.2%]
1–4 = Mild	174	18.9	[16.4%–21.6%]
5–7 = Moderate	396	42.9	[39.7%–46.2%]
8–10 = Severe	321	34.8	[31.7%–38.0%]
Skipped	12	1.3	
ER use (n = 922)			
Yes (sometimes or many times)	102	11.1	[9.1%–13.3%]
Skipped	20	2.1	
Chronic pelvic pain (n = 922)			
Yes	482	52.3	[49.0%–55.5%]
Skipped	10	1.1	

*Menstrual Cycle Characteristics.* The mean age at menarche was 11.9 years (SD ± 1.7; 95% CI: 11.8–12.0), ranging from 8 to 17 years. Early menarche (ages 8 to 10 years) was reported by 18.8% (95% CI: 16.7–21.4) of participants, while 9.5% (95% CI: 7.9–11.5) reported late menarche (ages 15 to 17 years). Irregular cycles were reported by 41.3% (95% CI: 38.4–44.4). Most participants characterized their menstrual flow as moderate (51.7%; 95% CI: 48.6–54.7) or abundant (38.4%; 95% CI: 35.4–41.4) (Table 1).

**Fig. 1:**
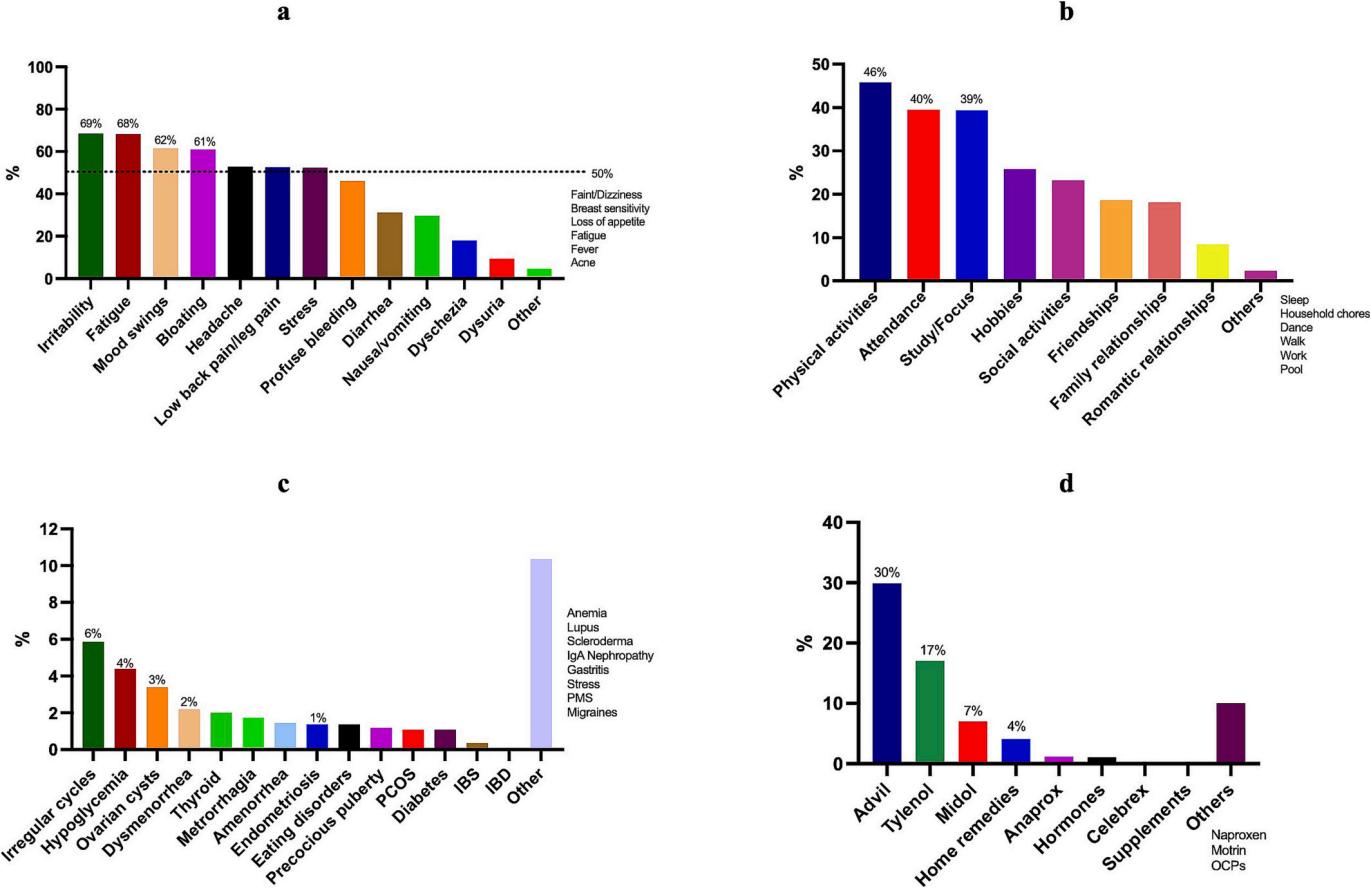
Menstrual cycle clinical profile of a cohort of 1094 Hispanic/Latina female adolescents living in Puerto Rico: menstrual symptoms (a), activities limited by symptoms (b), diagnosed conditions (c), Medications (d). PCOS – Polycystic Ovarian Syndrome, IBS – Irritable bowel syndrome, IBD – Inflammatory bowel disease, Thyroid (thyroid conditions, including hypo- and hyperthyroidism).

*Menstrual Cycle Clinical Profile*. Period-related pelvic pain was reported by 85.9% (95% CI: 83.6–87.9) of participants. Among those reporting pain, 44.3% (95% CI: 42.0–48.0) experienced moderate pain (NRS = 5–7) and for 36.4% (95% CI: 33.3–39.6) the pain was severe (NRS = 8–10). One in 10 (11.1%, 95% CI: 9.1%–13.3%) reported ER visits due to pelvic pain. When asked about the level of pelvic pain during their last menstrual period (LMP), 42.9% (95% CI: 39.7%–46.2%) indicated moderate pain, while 34.8% (95% CI: 31.7%–38.0%) indicated severe pain. Chronic pelvic pain (CPP), defined as pelvic pain persisting for six menstrual periods in a row or more, was reported by 52.3% (95% CI: 49.0%–55.5%) of participants **(**Table 1**)**. The most common menstrual symptoms were irritability (69.0%; 95% CI: 66.3–71.7), low energy or fatigue (68.0%; 95% CI: 65.2–70.8), mood swings (61.0%; CI: 58.1–63.9), and bloating (61.0%; 95% CI: 58.1–63.9). (Fig. 1A). The activities most affected by menstrual symptoms were physical activities (46.0%; 95% CI: 43.1–48.9), school attendance (40.0%; 95% CI: 37.1–42.9), and studying or concentrating (39.0%; 95% CI: 36.1–41.9) (Fig. 1B). Clinically diagnosed conditions reported by participants included irregular cycles (6.0%; 95% CI: 4.6–7.4), hypoglycemia (4.0%; 95% CI: 2.8–5.2), and ovarian cysts (3.0%; 95% CI: 2.0–4.0) (Fig. 1C). Medical diagnoses of dysmenorrhea (2.0%; 95% CI: 1.2–2.8) and endometriosis (1.0%; 95% CI: 0.4–1.6) were also reported. The use of over-the-counter (OTC) analgesics, such as ibuprofen or acetaminophen, was reported by 54.0% of participants (95% CI: 51.0–57.0). Only 1.0% (95% CI: 0.4–1.6) reported using hormonal treatments (Fig. 1D).

2) *Association of sociodemographic factors and menstrual health education features (having received menstrual education, sources of menstrual education, perceived understanding, trusted figures, level of comfort):*

**Fig. 2:**
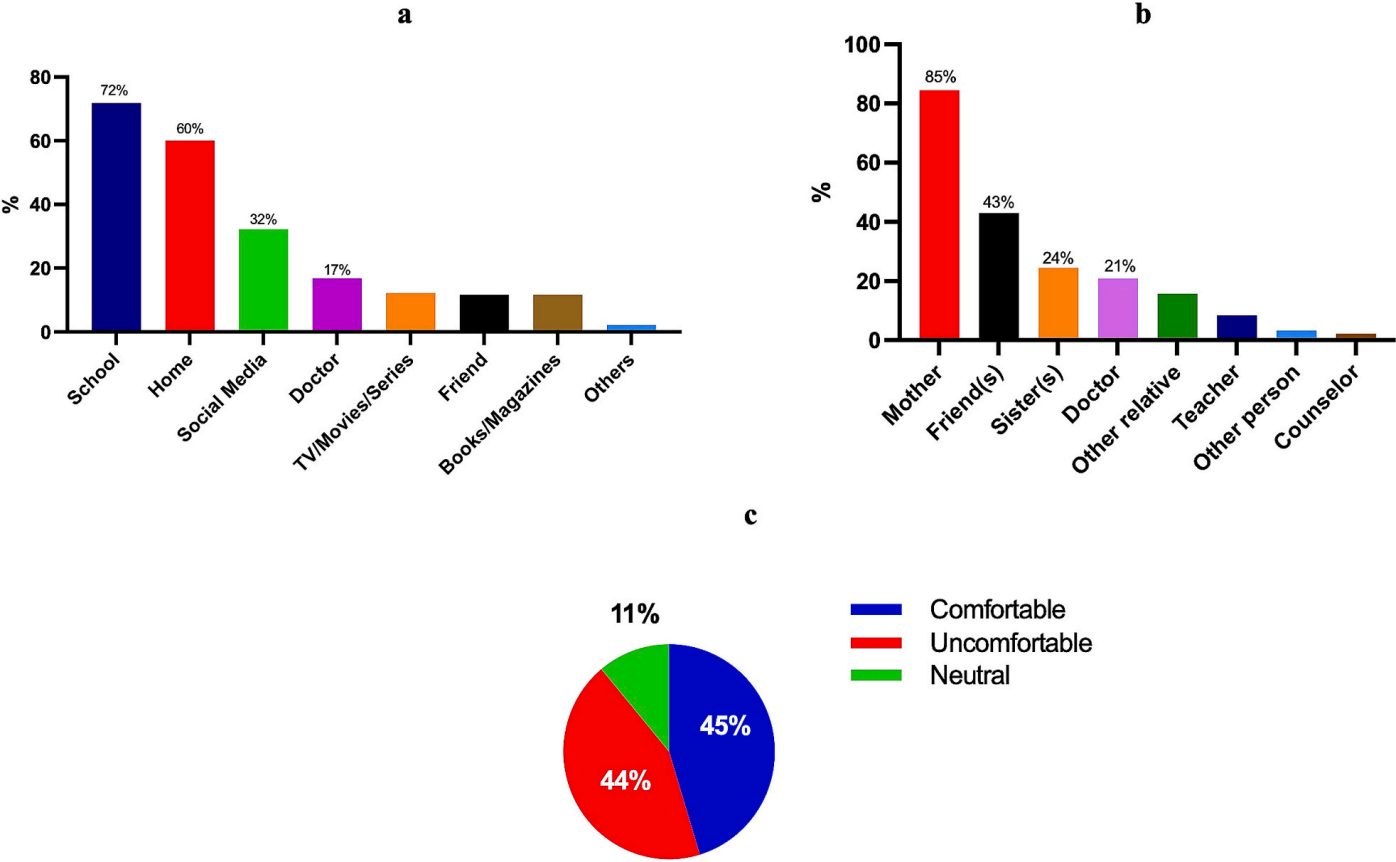
Menstrual Health Education: Sources (a), Trusted Figures (b) and Level of Comfort (c) among a cohort of 1094 Hispanic/Latina female adolescents living in Puerto Rico.

Most participants (89%; 95% CI: 87.1–90.9) reported having received menstrual cycle education. Overall, the main sources of education were schools (72.0%; 95% CI: 69.3–74.7), home (60.0%; 95% CI: 57.1–62.9), and social media (32.0%; 95% CI: 29.3–34.7), while doctors accounted for only 17% (95% CI: 14.8–19.2) (Fig. 2A). Mothers were the most trusted figures with whom they discussed menstrual health issues (85%; 95% CI: 82.9–87.1), followed by friends (43%; 95% CI: 40.1–45.9) and sisters (24%; 95% CI: 21.5–26.0) (Fig. 2B). While 45% (95% CI: 42.1–47.9) said they felt comfortable discussing menstrual health issues, 44% (95% CI: 41.1–46.9) expressed discomfort with the topic (Fig. 2C).

*Factors associated with having received menstrual education:* A logistic regression model was conducted to examine factors associated with having received education about menstrual health. The overall model was statistically significant (χ^2^ = 13.695, *p* = 0.008), although the explanatory power was relatively low (Nagelkerke R^2^ = 0.028). Residential zone was the only variable significantly associated with receiving menstrual health education, with participants from urban settings having higher odds of reporting menstrual education (OR = 2.67, 95% CI: 1.43–4.99, *p* = 0.002). Age, region, and type of school were not significantly associated with receiving menstrual cycle education (Table 2).

**Table 2:** Logistic regression results.

Variable	OR	95% CI	*P* value
Received menstrual health education			
Age	1.26	0.666–2.367	0.482
Residential zone	2.67	1.43–4.99	**0.002**
Region	1.02	0.644–1.626	0.923
Type of school	1.42	0.849–2.375	0.181
Sources of menstrual health education, School class			
Age	1.67	1.34–2.09	**<0.001**
Residential zone	1.64	1.14–2.35	**0.008**
Region	0.84	0.61–1.15	0.275
Type of school	1.83	1.28–2.62	**0.001**
Sources of menstrual health education, Mom or family member			
Age	0.76	0.62–0.93	**0.007**
Residential zone	1.00	0.74–1.34	0.987
Region	1.10	0.81–1.51	0.542
Type of school	0.81	0.60–1.11	0.187
Sources of menstrual health education, Friend			
Age	0.70	0.52–0.94	**0.020**
Residential zone	1.41	0.91–2.20	0.123
Region	1.36	0.89–2.10	0.158
Type of school	0.71	0.43–1.19	0.196
Sources of menstrual health education, TV/movies/documentaries			
Age	1.03	0.77–1.37	0.859
Residential zone	1.28	0.82–1.99	0.279
Region	1.09	0.71–1.66	0.702
Type of school	0.56	0.33–0.95	**0.031**
Sources of menstrual health education, Books/magazines/educational materials			
Age	1.15	0.86–1.53	0.346
Residential zone	1.51	0.98–2.32	0.064
Region	1.30	0.86–1.98	0.220
Type of school	0.34	0.18–0.64	**<0.001**
Sources of menstrual health education, Social media			
Age	1.46	1.19–1.79	**<0.001**
Residential zone	1.27	0.92–1.74	0.143
Region	1.32	0.98–1.79	0.068
Type of school	0.68	0.48–0.96	**0.029**
Sources of menstrual health education, Doctor			
Age	1.01	0.79–1.29	0.949
Residential zone	1.30	0.89–1.90	0.182
Region	1.35	0.94–1.94	0.105
Type of school	0.61	0.39–0.96	**0.032**
Perceived level of understanding			
Age	1.06	0.87–1.29	0.581
Residential zone	1.12	0.81–1.54	0.490
Region	0.78	0.58–1.05	0.102
Type of school	1.18	0.85–1.63	0.323
Preferred source of support, Mom			
Age	0.52	0.39–0.70	**<0.001**
Residential zone	0.93	0.60–1.45	0.751
Region	1.22	0.80–1.88	0.354
Type of school	0.81	0.52–1.26	0.356
Preferred source of support, Sister(s)			
Age	1.00	0.80–1.25	0.985
Residential zone	1.17	0.83–1.66	0.362
Region	1.27	0.92–1.77	0.147
Type of school	0.95	0.66–1.37	0.789
Preferred source of support, Teacher			
Age	0.91	0.65–1.28	0.588
Residential zone	1.29	0.77–2.16	0.333
Region	1.41	0.85–2.33	0.180
Type of school	1.11	0.63–1.93	0.724
Preferred source of support, Another family member			
Age	0.96	0.74–1.25	0.758
Residential zone	1.05	0.70–1.58	0.817
Region	1.25	0.85–1.84	0.259
Type of school	1.07	0.70–1.63	0.756
Preferred source of support, Medical provider			
Age	1.58	1.25–2.00	**<0.001**
Residential zone	1.18	0.82–1.68	0.376
Region	1.26	0.90–1.76	0.184
Type of school	0.62	0.41–0.93	**0.020**
Preferred source of support, Friend(s)			
Age	1.01	0.83–1.22	0.943
Residential zone	0.94	0.69–1.27	0.684
Region	1.18	0.88–1.57	0.267
Type of school	0.70	0.51–0.95	**0.024**
Preferred source of support, Counselor			
Age	0.85	0.46–1.55	0.590
Residential zone	0.63	0.21–1.90	0.412
Region	4.90	1.73–13.91	**0.003**
Type of school	1.33	0.41–4.29	0.636

Note. Bold P values indicate statistical significance (P < 0.05). “Age,” “Residential zone,” “Region,” and “Type of school” are the covariates included in each logistic regression model.

*Factors associated with the sources of menstrual health education:* The binary logistic regression analyses examining sources of menstrual health education showed that receiving information in a school class was significantly associated with older age (OR = 1.67, 95% CI: 1.34–2.09], *p* < 0.001), zone (OR = 1.64, 95% CI [1.14–2.35], *p* = 0.008), and type of school (OR = 1.83, 95% CI [1.28–2.62], *p* = 0.001). In contrast, obtaining menstrual health education at home from a mother or family member was negatively associated with age, indicating that older participants were less likely to report family-based education (OR = 0.76, 95% CI: 0.62–0.93, *p* = 0.007). Similarly, older participants had lower odds of receiving information from a friend (OR = 0.70, 95% CI: 0.52–0.94, *p* = 0.020). Public school was significantly associated with lower odds of reporting television, movies, series, or documentaries as information sources (OR = 0.56, 95% CI: 0.33–0.95, *p* = 0.031), as well as books, magazines, or educational materials (OR = 0.34, 95% CI: 0.18–0.64, *p* < 0.001). Social media use as a source of menstrual health education was positively associated with age (OR = 1.46, 95% CI: 1.19–1.79, *p* < 0.001) but inversely associated with type of school (OR = 0.68, 95% CI: 0.48–0.96, *p* = 0.029). Finally, public school was also significantly associated with lower odds of reporting physicians as a source of menstrual health education (OR = 0.61, 95% CI: 0.39–0.96, *p* = 0.032) (Table 2).

*Factors associated with comfort discussing menstrual health topics:* A logistic regression model was performed to evaluate factors associated with feeling comfortable discussing menstrual health topics with others. The overall model was statistically significant (χ^2^ = 46.41, *p* < 0.001) and explained between 8.1% and 13.3% of the variance in the outcome. Older age was significantly associated with greater comfort discussing menstrual health [OR = 3.13, 95% CI: 2.12–4.62, p < 0.001]. Public school participants were less likely to feel comfortable although this did not reach statistical significance [OR = 0.58, 95% CI:0.34–1.01, *p* = 0.052]. Region of residence was not significantly associated with comfort discussing these topics [OR = 1.04, 95% CI:0.62–1.75, *p* = 0.887], nor was residential zone [OR = 0.76, 95% CI:0.44–1.29, *p* = 0.30] (Table 2).

*Factors associated with perceived level of understanding of menstrual health topics:* The binary logistic regression model examining participants' understanding of the menstrual cycle was not statistically significant overall, indicating that the predictors included in the model did not meaningfully explain differences in menstrual cycle understanding, χ^2^(4) = 6.13, *p* = 0.189. None of the independent variables, including age group (OR = 1.06, 95% CI: 0.87–1.29], *p* = 0.581), region (OR = 0.78, 95% CI: 0.58–1.05], *p* = 0.102), zone (OR = 1.12, 95% CI: 0.81–1.54, *p* = 0.490), or type of school (OR = 1.18, 95% CI: 0.85–1.63, *p* = 0.323), were significantly associated with reporting a good understanding of the menstrual cycle. These findings suggest that menstrual cycle comprehension did not differ significantly according to demographic or school-related characteristics in this sample (Table 2).

*Factors associated with trusted figures for discussing menstrual health topics:* The binary logistic regression analyses examining trusted figures for discussing menstrual health topics showed several significant associations. Reporting mothers as a trusted figure was significantly associated with age group, with older participants showing lower odds of identifying their mother as someone they trust to discuss menstrual health topics (OR = 0.52, 95% CI: 0.39–0.70, *p* < 0.001). No significant associations were found for reporting sisters, teachers/professors, or other family members as trusted figures, as all predictors were non-significant (all *p* > 0.05). Participants were more likely to identify physicians as trusted figures with increasing age (OR = 1.58, 95% CI: 1.25–2.00, p < 0.001). Public school was associated with lower odds of reporting physicians (OR = 0.62, 95% CI: 0.41–0.93, *p* = 0.020) and friends (OR = 0.70, 95% CI: 0.51–0.95, *p* = 0.024) as trusted sources for menstrual health discussions. Finally, metro region was significantly associated with identifying counselors as trusted figures (OR = 4.90, 95% CI:1.73–3.91, *p* = 0.003) (Table 2).

3) *Association of sociodemographic factors with menstrual pain and ER visits due to pelvic pain or menstrual symptoms:*

*Factors associated with reporting moderate to severe menstrual pain*: Binary logistic regression analysis was conducted to examine the association between sociodemographic factors and reporting moderate to severe menstrual pain compared with mild pain. The overall model was not statistically significant (χ^2^ = 4.335, *p* = 0.363), indicating that the predictors included in the model did not significantly explain variation in menstrual pain severity. None of the variables examined were significantly associated with reporting moderate/severe pain, including age (OR = 1.27, 95% CI: 0.96–1.69, *p* = 0.095), region (metro vs. non-metro) (OR = 1.10, 95% CI: 0.73–1.65, *p* = 0.662), geographic zone (OR = 1.30, 95% CI: 0.82–2.05, *p* = 0.266), or type of school (OR = 1.20, 95% CI: 0.78–1.85, *p* = 0.406). These findings suggest that the sociodemographic characteristics evaluated were not significantly associated with menstrual pain severity in this sample (Table 2).

*Factors associated with ER utilization*: Binary logistic regression analysis was used to assess factors associated with ER visits due to pelvic pain or menstrual-related symptoms. The model was statistically significant (χ^2^ = 315.093, *p* < 0.001) and explained between 32.2% and 43.0% of the variance according to the Cox & Snell and Nagelkerke pseudo-R^2^ statistics. Older age was significantly associated with a higher likelihood of ER visits (OR = 1.70, 95% CI: 1.29–2.24, p < 0.001). Public school participants were less likely to utilize the ER than their private school counterparts. While participants from non-metropolitan regions were more likely to report ER visits compared with those from metropolitan areas (OR = 1.48, 95% CI: 1.02–2.16, *p* = 0.040), living on an urban vs. a rural residential zone was not significantly associated with ER visits (OR = 0.81, 95% CI: 0.54–1.21, *p* = 0.309) (Table 2).

## 4 Discussion

This study was designed to address the lack of data on menstrual health profiles and menstrual cycle education gaps among menstruating adolescents in Puerto Rico. These findings are crucial to informing healthcare providers, teachers, and policymakers about the specific challenges and knowledge gaps faced by adolescent populations. We identified high rates of menstrual irregularities, distress and pelvic pain, along with variations in education, trusted figures, comfort and ER utilization regarding menstrual health based on factors such as age, school type, region or residential zone. Our results indicate the need for developing tailored menstrual health education initiatives that are informed by research.

The menstrual profile of our cohort is characterized by a predominance of regular cycles with moderate to heavy menstrual flow. The average age at menarche, 12 years old, is consistent with other cohorts around the globe. However, we observed a higher rate of early menarche (<11 years old) compared to previous reports from the US (20% vs. 10%) [18], [19]. A significant finding is the high prevalence of severe menstrual pelvic pain (reported by 36% of our study participants), causing 1 in 10 to seek ER care. Our study aligns with other reports of high levels of dysmenorrhea in female adolescents, most commonly primary dysmenorrhea, which often normalizes with time [5], [9], [20], [21]. However, it is also possible that some of our study participants may have endometriosis, a common cause of secondary, severe, and refractory dysmenorrhea in female adolescents [2], [22]. Irregular cycles were also highly prevalent in our cohort, which are a common finding near menarche; however, menstrual irregularities may also potentially indicate underlying conditions such as polycystic ovary syndrome (PCOS) or hormonal imbalances [23]. Symptom management among participants was suboptimal, with a reliance on OTC medications and infrequent use of hormonal treatments, such as OCPs, which have been proven to be highly effective for the management of period pain and PCOS. Self-reported clinical diagnoses of primary dysmenorrhea, menorrhagia, endometriosis and PCOS were notably low, suggesting insufficient medical intervention despite the high prevalence of severe symptoms. These results could indicate a lack of awareness about available hormonal treatments and a delayed urgency in seeking medical attention for menstrual-related symptoms, which would need to be investigated in follow-up research. As suggested in previous studies, our results may reflect a concerning and widespread perception that menstrual pain is normal, except among those reporting higher pain scores [9]. Therefore, this study supports the importance of addressing menstrual education in schools and at home to help menstruating adolescents manage their menstrual health effectively and seek timely medical attention, potentially preventing negative impacts on their overall health and academic performance [24].

The participants in the study reported a high incidence of symptoms associated with their menstrual cycle beyond dysmenorrhea and CPP; the most common included mood swings, fatigue/low energy, nausea, and bloating. The data illustrate the negative impact and significant burden of menstrual symptoms on daily activities such as studying, school attendance and sports for a high proportion of participants, as shown in other studies [5], [9], [16]. Menstrual education for this demographic should include strategies, such as lifestyle modifications and stress management techniques to enhance overall well-being and daily functioning. Symptoms like irritability, mood swings and stress may indicate disorders including PMS and Premenstrual dysphoric disorder (PMDD). Thus, it is important to increase awareness of these conditions and help individuals recognize when symptoms are severe enough to warrant medical intervention [25], [26]. As stated by Armour and colleagues [9], improving menstrual health literacy is vital for encouraging health-seeking behaviors among this population and their caregivers [9].

Most participants in this study reported receiving menstrual health education primarily at school, followed by their home, social media, and, less frequently, physicians. These results show the fundamental role schools play in educating about the menstrual cycle, its symptoms, and related conditions. Our findings support the importance of collaborating with educational systems to ensure that menstrual health curricula provide clear and accurate information about both what is normal in the menstrual cycle, and what may indicate the need for medical intervention. Additionally, the findings demonstrate the strong influence of social media as a source of information, highlighting the need to create and disseminate evidence-based educational content. Participants in our study typically trusted their mothers the most, followed by friends and sisters, as expected, given these individuals' close roles in their personal environment [27]. Therefore, while schools bear a societal responsibility of educating youth about the developmental processes of puberty, mothers and caregivers must also strive to improve their menstrual health literacy to better support this population.

The next aim of our study involved gaining additional insights into the variables that may influence receiving menstrual health education, levels of understanding, comfort in discussing these topics, and trusted figures. Most participants reported receiving their menstrual education from school, with slight differences among age groups. Participants living in urban settings were more likely to receive menstrual health education, but we observed no differences by age, type of school or region.

Younger participants were more likely to receive menstrual health education at home, trusted their mothers more, and felt less comfortable talking about these topics. However, they did not show significantly lower levels of perceived understanding compared to older participants. Older participants were more likely to trust their doctors and were more likely to use social media as a source of menstrual health education, while the youngest relied more on their close circle (mothers, friends). These data are consistent with our observation that older participants were more likely to report feeling comfortable discussing menstrual health topics and may reflect that young adults visit a gynecologists more commonly than younger teens.

Differences in sources of information, trusted figures and comfort between participants from public and private schools may be attributed to variations in menstrual education delivery and content. For instance, many private schools are faith-based, and their curricula may differ from those of public schools. Regardless of school type, our study revealed that the education system plays a crucial role in promoting health education about the menstrual cycle, as they are often the first and most consistent source of structured health education. By providing early, accurate, and age-appropriate information about the menstrual cycle, school health curricula can foster an understanding of normal biological processes, recognition of potential menstrual health issues, and open discussions about reproductive health. Effective menstrual education should not only cover proper hygiene and cycle tracking but also raise awareness of the impact of lifestyle and behaviors on menstrual health, including menstrual distress, empowering students with knowledge and self-care skills. Moreover, schools should address the social and emotional aspects of menstruation by reducing stigma, normalizing conversations, and creating a safe space where teens feel comfortable asking questions and seeking support, especially those who may feel embarrassed or isolated due to limited knowledge [28]. Beyond schools, our study suggests the potential effectiveness of involving parents, guardians, and peers in menstrual education, underscoring the need for further exploration of this approach.

Differences based on the participants' place of residence (metropolitan or urban areas vs. small towns or rural areas) may reflect the greater availability of resources in metropolitan regions. In these areas, reduced stigma around seeking gynecological care for menstrual health concerns may facilitate access to medical appointments. Trust in mothers was the highest across all participants, regardless of where they lived. However, other notable differences, such as the higher likelihood of urban residents to receive menstrual health education and of participants in rural areas to rely on media sources (both traditional and social) for obtaining menstrual health education, warrant further research to better understand these behaviors.

Binary logistic regression models showed that reporting moderate to severe menstrual pain was not associated with any of the sociodemographic variables examined, suggesting that the burden of severe menstrual pain does not vary across age, residential area, and proxies of socioeconomic status such as school type. This pattern supports the need for universal screening and systematic assessment of menstrual pain in adolescents across sociodemographic groups. ER visits were significantly associated with older age, private school attendance, and residency in non-metropolitan areas. This pattern may reflect differences in access to and navigation of care, whereby older adolescents have more agency to seek emergency services independently, students in private schools may be more likely to have private insurance or higher socioeconomic resources that facilitate ER use, and those living in non-metropolitan areas may have fewer gynecologic care options, leading them to rely on emergency departments for acute menstrual symptoms.

This study revealed a high prevalence of severe menstrual symptoms, cycle irregularities and menstrual distress among over 1000 participants encompassing all regions of Puerto Rico, with broad inclusion of middle school, high school, and college-aged menstruating adolescents and young women from diverse settings (public and private schools, urban and rural areas, large cities, and small towns). However, the generalizability of its findings is limited due to the non-probabilistic recruitment strategies used that included flyer distribution using social media and access to internet/smartphone to answer the survey. Although the large sample size and geographic diversity strengthen the study, the results should not be interpreted as population-level prevalence estimates for all Puerto Rican adolescents and are most appropriately considered representative of the recruited sample. While the reliance on self-reported data using an online, self-administered survey may introduce recall bias or inaccuracies, particularly with sensitive and often misunderstood topics like menstrual health, the study successfully achieves its objective of generating the first comprehensive menstrual health profile of Puerto Rican menstruating adolescents. Follow up studies include assessments of menstrual health literacy using validated instruments, investigations into health-seeking behaviors, time-to-care and barriers to diagnosis for gynecologic conditions, and qualitative investigations into the experiences, perceptions and emotions around the menstrual cycle in menstruating adolescents from Puerto Rico and other Hispanic/Latina populations in the US and Latin America.

In summary, the findings of this cross-sectional study provide crucial data for medical professionals, relevant government ministries (health and education), and policymakers regarding the specific needs and challenges of menstruating adolescents, a population that is underrepresented in research studies. Our research highlights the importance of incorporating teens' sociodemographics into the development of menstrual health curricula, aiming to increase awareness of menstrual-related conditions and improve their overall reproductive health and wellbeing.

## Data sharing

De-identified data will be available in aggregate to protect participants confidentiality. Individual level data will not be shared as this study was conducted on minors. Analysis code including summary tables and syntax will be shared under IRB approved protocols.

## Sources of funding/acknowledgements

This study was conducted in part thanks to support from *Fundación Intellectus*, Ponce, Puerto Rico, who had no role in study design, data collection or analysis, writing this manuscript or decision to submit for publication. Thanks to the Department of Education of Puerto Rico for dissemination of the study survey and to medical students Amanda Detrés, María A. Lopera, Ariana De Jesús, Nashalie Ortiz, and Valeria Bracero for clinical insights on the study results.

## CRediT authorship contribution statement

**Idhaliz Flores-Caldera:** Writing – review & editing, Writing – original draft, Visualization, Validation, Supervision, Resources, Project administration, Methodology, Investigation, Funding acquisition, Formal analysis, Data curation, Conceptualization. **Kathleen N. Morales:** Visualization, Methodology, Investigation, Formal analysis, Data curation. **Braulio I. Rivera:** Visualization, Methodology, Investigation, Formal analysis, Data curation. **Maricarmen Colón-Díaz:** Writing – review & editing, Writing – original draft, Validation, Formal analysis, Data curation, Conceptualization. **Yeidelin Nieves:** Writing – review & editing, Writing – original draft, Investigation, Formal analysis. **Christy Rosado:** Methodology, Investigation, Formal analysis, Data curation, Conceptualization. **Maria F. Martínez:** Writing – review & editing, Writing – original draft, Investigation. **Adriana Diaz-Betancourt:** Writing – original draft, Investigation, Formal analysis. **Angela Cooke-Jackson:** Methodology, Investigation, Conceptualization. **Valerie Rubinsky:** Methodology, Investigation, Conceptualization.

## Declaration of competing interest

The authors declare the following financial interests/personal relationships which may be considered as potential competing interests: Dr. Idhaliz Flores-Caldera is co-owner and Scientific Advisor of Sur 180 Therapeutics, and Chief Scientific Officer of Nura Health. All other authors declare no potential conflicts of interest with respect to the research, authorship, and/or publication of this article.
