# Supplementary material for: Characterizing menstrual health and education needs among menstruating adolescents: A cross-sectional study on dysmenorrhea, cycle irregularities, and menstrual education
Source: Dialogues Health. 2026 Jul 27;9:100327. doi: 10.1016/j.dialog.2026.100327 (PMC13476648; doi:10.1016/j.dialog.2026.100327)
Supplement: Supplementary file 1 [file mmc1.pdf]

English version

Study title: ***Study on menstrual healthand educationin adolescentsa and young women in Puerto Rico***

Introduction:

Hi!

We invite you to participate in a study on the menstrual health of Puerto Rican adolescents and young women. Your participation consists of answering questions anonymously about your menstrual cycle and related conditions and where do you get menstrual cycle information.

Answering this questionnaire is voluntary. You don't have to answer all the questions and you can stop answering them at any time.

Thank you for taking your time to help us learn more about menstrual health in Puerto Rican girls!

1. Age: _______ (add ages 11 – 20)
2. Grade/ University or Institute year: _______ (add High School grades, University/Institute) → What university/institute? __________
3. Type of High school you attend or attended: ☐ public ☐ private ☐ homeschool ☐ free studies/certificate
4. City/Town: ___________________ Zone: ☐Urban ☐Rural
5. At what age did you have your first period? _____years old (add ages 8 – 20)
6. Your period is: ____ Regular ____ Irregular ___ Don't know
7. How often do you have your period on average? ______ days (example, every 28 days) (If it is too irregular to say exactly, write "irregular")
8. How many days do you bleed during your menstrual cycle on average? ____ days (example, for 5 days)
9. Do you use a mobile app to monitor your menstrual cycle? ☐ Yes ☐ No

   If Yes, which one?
10. The following figure shows examples of how much bleeding you might have **__every 4 hours__** during your period. Describe the amount of bleeding you usually experience during your period on days when it's **heaviest:**

    ☐ light ☐ mild ☐ moderate ☐ heavy

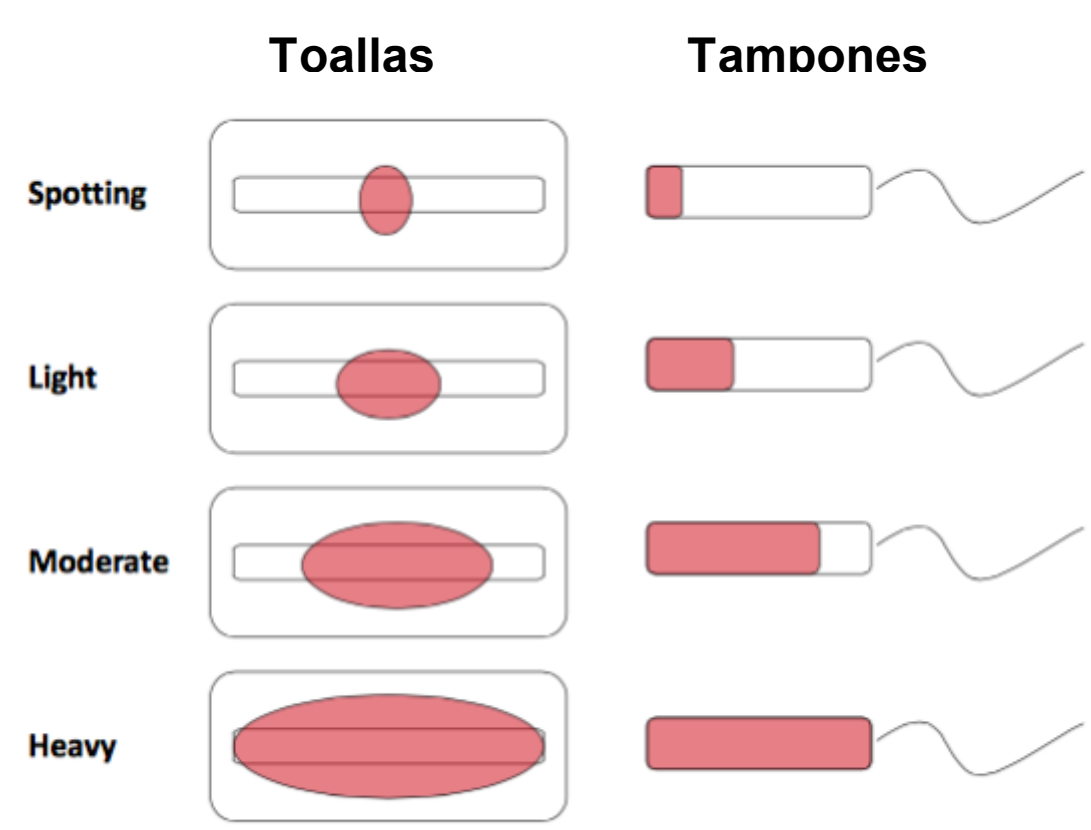

11. Do you suffer from pelvic pain during menstruation? (Pain in the triangle area in the figure)

    ☐ No → go to **question 17**

    ☐ Yes, always →

    ☐ Yes, sometimes→

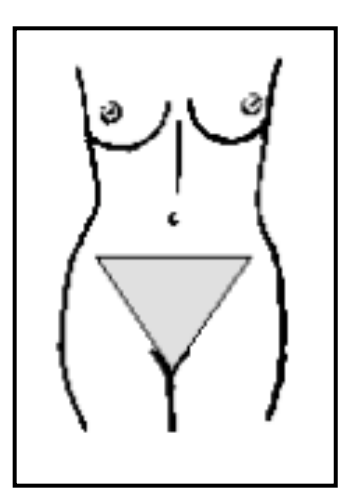

12. How do you describe the pelvic pain?

    ☐ mild cramps (I don't need to take pain medication)

    ☐ moderate cramping (I usually have to take pain medication)

    ☐ severe cramps (I have to take pain medication and rest)
13. When do you experience the pain in general?

    ☐ The first day of my period

    ☐ The first and second day of my period

    ☐ Days before the period

☐ When I'm ovulating

☐ All the time

14. At what age did you start suffering from pain with menstruation? __________years

15. Have you experienced pelvic pain for more than 6 menstrual cycles in a row? ☐ Yes ☐ No

16. How severe was the pelvic pain **when it was worse** during **your last menstrual period?** *Use the scale from 0 to 10, where 0 = no pain and 10 = the worst pain you can imagine.*

0	1	2	3	4	5	6	7	8	9	10
**No dolor**										**El peor dolor imaginable**

17. Have you had to visit an emergency room or emergency room because of pelvic pain or other symptoms you experience during your period?

☐ Yes, many times

☐ Yes, sometimes

☐ No

18. Which of the following symptoms do you experience during your menstruation? (check all that apply)

☐ I have no symptoms ☐ Pain when having a bowel movement ☐ Painful urination ☐ Nausea/vomiting

☐ Heavy bleeding ☐ Bloating ☐ Nausea/vomiting ☐ Diarrhea/intestinal upset

☐ Lower back and leg pain ☐ Headache ☐ Low tiredness/energy

☐ Irritability or mood swings ☐ Stress ☐ Change in emotions ☐ Other: ____

19. Have you visited a doctor because of these symptoms?

☐ No ☐ Yes → What kind of doctor? (gynecologist, pediatrician, emergency, general practitioner, gastroenterologist, other) (check all that apply)

20. Are you taking medication for these symptoms?

☐ No ☐ Yes → Which ones? ___(check all that apply)

☐ Midol. ☐ Advil ☐ Tylenol ☐ Anaprox ☐ Celebrex ☐ Hormones. ☐ Supplements

☐ Herbal teas. ☐ Other: ________

21. Do you think the treatments you take work for you?

☐ No ☐ Yes

22. Do you consider that the symptoms you experience during your menstruation limit you in your daily activities?

☐ No ☐ Yes → If yes, in which ones? (check all that apply)

☐ Attend school ☐ Playing sports/physical activities ☐ Participating in social activities

☐ Relationship with my family ☐ Relationship with my friends ☐ Relationship with my partner (If applicable)

☐ Study/Focus on tasks ☐ Hobby ☐ Other: ____ (Which other?)

23. Has a health professional ever diagnosed you: (check all that apply)

☐ Endometriosis

☐ Polycystic ovaries (PCOS)

☐ Thyroid

☐ Precocious puberty

☐ Cysts in the ovaries

☐ Irregular cycles (menstruation does not come every month)

☐ Dysmenorrhea (pain with menstruation)

☐ Profuse bleeding (metrorrhagia)

☐ Menstruation stopped (amenorrhea)

☐ Eating disorders (bulimia, anorexia)

☐ Irritable bowel syndrome (IBS)

☐ Inflammatory colon disease (IBD)

☐ Diabetes

☐ Hypoglycemia

☐ Other: → Which other diagnosis?

24. For each diagnosis selected, at what age were you diagnosed? ____ (years)

25. Does anyone in your family have endometriosis? (a painful disease that causes severe menstrual pain)

    ☐ No ☐ I Don't Know ☐ Yes → If yes, who? (mom, sisters, cousins, aunts, grandmothers)

26. Have you received menstrual cycle education?

    ☐ No ☐ Yes →

27. Where did/do you get menstrual cycle education from? (check all that apply)

    ☐ in a school class ☐ from my mom ☐ from a relative ☐ from a friend

    ☐ on TV, movies, documentaries, series ☐ from books/magazines/educational material

    ☐ from social media (Instagram, Facebook, TikTok) ☐ Other: __________

28. Do you feel that you have a good understanding of the menstrual cycle?

    ☐ Yes, I have received sufficient information about the menstrual cycle

    ☐ I have received some information about the menstrual cycle, but I still have questions

    ☐ Not at all, I have not received sufficient information about the menstrual cycle and I have many questions

29. Who do you trust if you have questions about your menstrual cycle, your period or any related topic?

    ☐ my mom ☐ sister(s) ☐ family member ☐ teacher/professor ☐ Doctor ☐ Friend(s) ☐ Counselor

    ☐ another person: _____________

30. How comfortable you feel talking to others about the menstrual cycle, the period and related topics?

    ☐ Very comfortable ☐ Somewhat comfortable ☐ Neutral ☐ Somewhat uncomfortable ☐ Very uncomfortable

31. Do you have any questions or comments on the topic of the menstrual cycle for the study team?

32. Would you be willing to participate in a follow-up study to find the best strategies to educate Puerto Rican teens about the menstrual cycle? If you are, send us an email to endopr@gmail.com.

Final thanks

Thank you for participating! These results will help us understand how common gynecological conditions are in Puerto Rican adolescents and to develop educational programs on menstrual health in Puerto Rico's high schools.

Spanish version

Título del estudio: *Estudio sobre salud menstrual y educación en salud menstrual en adolescentes y mujeres jóvenes de Puerto Rico*

Introducción:

Hola!

Te invitamos a participar en un estudio sobre la salud menstrual de adolescentes y mujeres jóvenes puertorriqueñas. Tu participación consiste en contestar unas preguntas de forma anónima sobre tu ciclo menstrual y condiciones relacionadas, y sobre dónde consigues información sobre el ciclo menstrual.

Contestar este cuestionario es algo **voluntario**. No tienes que contestar todas las preguntas y puedes dejar de contestarlas en cualquier momento.

Gracias por darnos tu tiempo para conocer más sobre salud menstrual en chicas puertorriqueñas!

1. Edad: _______ (añadir edades 11-20)
2. Grado/Año de Universidad o Instituto: _______ (añadir grados, Universidad/Instituto) → Qué Universidad/Instituto? _________
3. Tipo de escuela superior a la que asistes (o asististe): ____ pública ____ privada __ homeschooling ___ estudios libres (módulos)
4. Pueblo/Ciudad: ____________________ Zona: ____ rural (campo) ___ urbana (pueblo)
5. ¿A qué edad tuviste tu primera menstruación? _____ años (añadir opciones 8 - 20)
6. ¿Sabes si tu menstruación es: ____ Regular ____ Irregular?
7. ¿Cada cuántos días tienes tu menstruación en promedio? ______ días (ejemplo, cada 28 días) Si es muy irregular para decir # exacto escribe "irregular"
8. ¿Cuántos días sangras durante tu período menstrual en promedio? ____ días (ejemplo, por 5 días)
9. ¿Usas una aplicación móvil para monitorear tu ciclo menstrual? ☐ Sí ☐ No

   Sí -> Cual app? ________

10. La siguiente figura muestra ejemplos sobre la cantidad de sangrado que podrías tener **cada 4 horas** durante tu período. Describe la cantidad de sangrado que usualmente experimentas durante tu período en los días en que éste es **más abundante:**

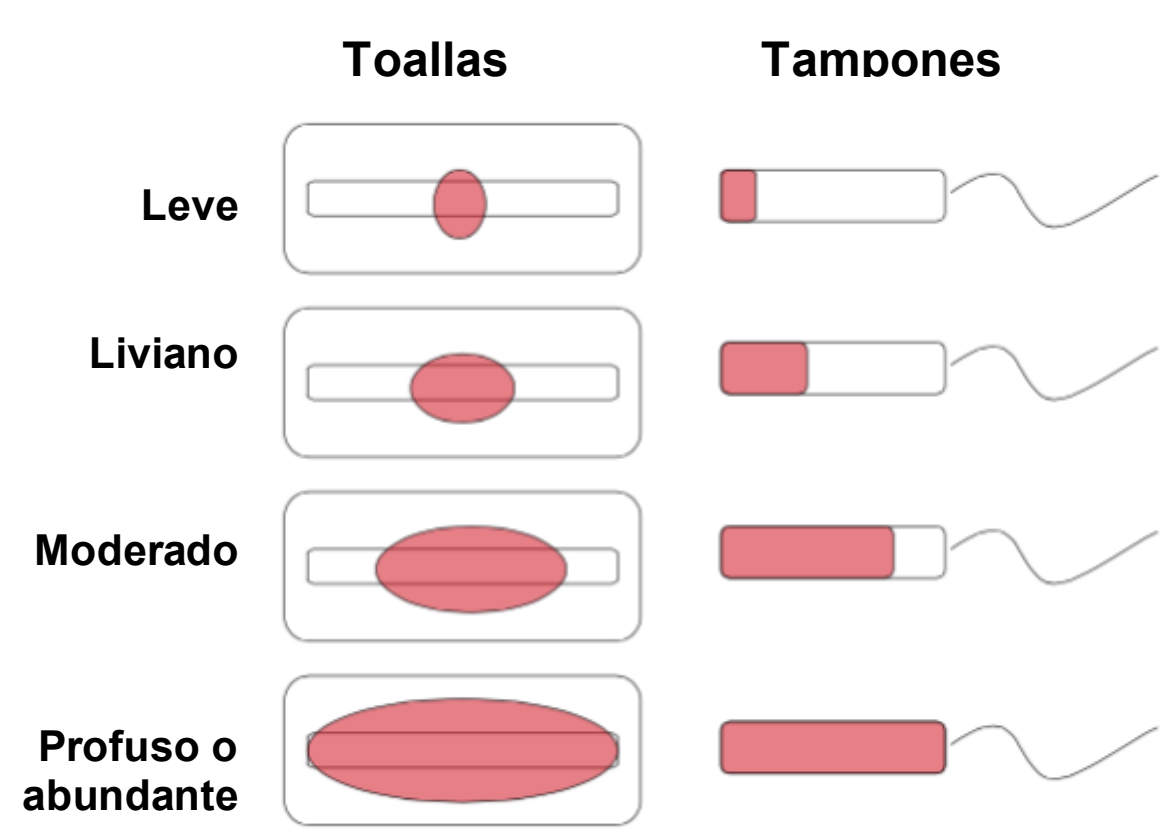

   ☐ leve ☐ liviano ☐ moderado ☐ abundante

11. ¿Sufres de dolor pélvico durante la menstruación? (Dolor en el área del triángulo en la figura)

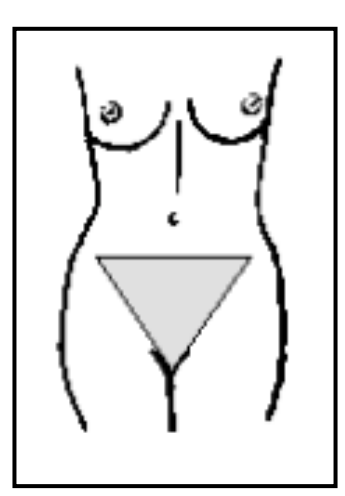

   ☐ No → Pasa a la **pregunta 17**

   ☐ Sí, a veces →

   ☐ Sí, siempre →

12. ¿Cómo describes ese dolor?

   ☐ calambres o dolor leve (no necesito tomar medicamentos para el dolor)

   ☐ calambres o dolor moderados (usualmente tengo que tomar medicamentos para el dolor)

   ☐ calambres o dolor severos (tengo que tomar medicamentos para el dolor y descanso)

13. ¿Cuándo sientes ese dolor?

   ☐ el primer día de mi período

☐ el primer y segundo día de mi período

☐ días antes del período

☐ cuando estoy ovulando

☐ todo el tiempo

14. ¿A qué edad comenzaste a sufrir de dolor con la menstruación? ___________ años

15. ¿Has experimentado dolor pélvico por más de 6 ciclos menstruales corridos? ☐ Sí ☐ No

16. ¿Cuán severo era el dolor pélvico **cuando era peor** durante **tu último período menstrual**. *Usa la escala del 0 al 10, donde 0 = no dolor y 10 = el peor dolor que puedas imaginar.*

0	1	2	3	4	5	6	7	8	9	10
**No dolor**										**El peor dolor imaginable**

17. ¿Has tenido que visitar una sala de emergencias o urgencias por el dolor pélvico u otros síntomas que experimentas durante tu menstruación?

☐ Sí, muchas veces

☐ Sí, algunas veces

☐ No

18. ¿Cuál de los siguientes síntomas experimentas durante tu menstruación? (marca todas las que apliquen)

☐ No tengo síntomas ☐ Dolor al evacuar ☐ Dolor al orinar ☐ Náuseas/vómitos

☐ Sangrado profuso ☐ Inflamación del abdomen ☐ Náuseas/vómitos ☐ Diarreas/malestar intestinal

☐ Dolor espalda baja y piernas ☐ Dolor de cabeza ☐ Cansancio/energía baja

☐ Irritabilidad o cambios de humor ☐ Estrés ☐ Cambio en emociones ☐ Otro: _____________

19. ¿Has visitado a algún médico debido a estos síntomas?

☐ No ☐ Sí → ¿Qué tipo de médico? (marca todas las que apliquen)

☐ ginecológo/a ☐ pediatra ☐ emergencias ☐ médico generalista ☐ gastroenterólogo

☐ otro:_____

20. ¿Estás tomando medicamentos para estos síntomas?

☐ No ☐ Sí →¿Cuáles? (marca todas las que apliquen)

☐ Midol ☐ Advil ☐ Tylenol ☐ Anaprox ☐ Celebrex ☐ Suplementos
☐ Teses ☐ Hormonas ☐ Otros: __________

21. ¿Consideras que los tratamientos que tomas te funcionan?

☐ No me alivian ☐ Sí, un poco ☐ Sí, bastante ☐ Sí, mucho

22. ¿Consideras que los síntomas que experimentas durante tu menstruación te limitan en tus actividades diarias?

☐ No ☐ Sí → ¿En cuáles? (marca todas las que apliquen)

☐ Asistir a la escuela ☐ Practicar deportes /actividades físicas ☐Participar en actividades sociales

☐ Relación con mi familia ☐ Relación con mis amistades ☐Relación con mi pareja (Si aplica)

☐ Estudiar/Concentrarme en tareas ☐ Practicar hobbies ☐ Otras: ______ → Cuál otra? ________

23. ¿Alguna vez un profesional de la salud te ha diagnosticado: (marca todos los que apliquen)

☐ Endometriosis

☐ Ovarios poliquísticos (PCOS)

☐ Enfermedad de la tiroides

☐ Pubertad precoz

☐ Quistes en los ovarios

☐ Ciclos irregulares (la menstruación no llega todos los meses)

☐ Dismenorrea (dolor con la menstruación)

☐ Sangrado profuso (metrorrhagia)

☐ Se detuvo la menstruación (amenorrhea)

☐ Desórdenes alimenticios (bulimia, anorexia)

☐ Síndrome de colon irritable (IBS)

☐ Enfermedad inflamatoria del colon (IBD)

☐ Diabetes

☐ Hipoglicemia

☐ Otra condición: ________________

24. ¿A qué edad te diagnosticaron? _____ (años)

25. ¿Alguien en tu familia tiene endometriosis?

☐ No ☐ No sé ☐ Sí → (mamá, hermanas, primas, tías, abuelas)(marca todas las que apliquen)

26. ¿Has recibido educación sobre el ciclo menstrual?

☐ No ☐ Sí →

27. ¿En dónde recibiste educación sobre el ciclo menstrual? (marca todas las que apliquen)

☐ en una clase en la escuela ☐ de mi mamá ☐ de un familiar ☐ de amiga(s)

☐ en la TV, películas, documentales, series ☐ en libros/revistas/material educativo

☐ en redes sociales (Instagram, Facebook, TikTok) ☐ Otro:____________

28. ¿Piensas que tienes una buena comprensión del ciclo menstrual?

☐ Sí, he recibido suficiente información sobre el ciclo menstrual

☐ Sí, he recibido información sobre el ciclo menstrual, pero todavía tengo preguntas o dudas

☐ No he recibido suficiente información y tengo muchas preguntas/dudas

29. ¿En quién confías si tienes preguntas sobre tu ciclo menstrual, tu período o cualquier tema relacionado?

☐ mamá ☐ hermana(s) ☐ otro familiar ☐ maestra/profesora ☐ médico ☐ amiga(s) ☐ consejero/a

☐ Otra persona: ______________

30. ¿Qué tan cómoda te sientes hablando con otras personas sobre el ciclo menstrual, el período y temas relacionados?

☐ Muy cómoda ☐ Bastante cómoda ☐ Neutral ☐ Bastante incómoda ☐ Muy incómoda

31. ¿Tienes alguna pregunta o comentario sobre el tema del ciclo menstrual para el equipo del estudio?

32. ¿Estarías dispuesta a participar en un estudio de seguimiento para encontrar las mejores estrategias para educar a adolescentes puertorriqueñas sobre el ciclo menstrual? **Si lo estás, envíanos un email a endopr@gmail.com**.

Agradecimiento final:

Gracias por participar! Estos resultados nos ayudarán a entender cuán comunes son las condiciones ginecológicas en adolescentes puertorriqueñas y a desarrollar programas educativos sobre salud menstrual en las escuelas superiores

de Puerto Rico.

FLYER CONTENT

(A flyer will be developed in collaboration with the PRI Public Relations office and will be submitted to IRB review once is ready)

ARE YOU BETWEEN 11 AND 20 YEARS OLD...?

YOU COULD PARTICIPATE IN A STUDY ON THE MENSTRUAL CYCLE

We are recruiting Puerto Rican young women and adolescents to participate in a study that will help us understand the characteristics of the menstrual cycle, the prevalence of conditions associated and the sources of information used to educate themselves on this topic.

THIS STUDY IS ANONYMOUS.

It will only take about 5 minutes to answer the questions.

If you have questions, please contact the study's Principal Investigator

Dr. Idhaliz Flores Caldera

iflores@psm.edu

Endopr@gmail.com

787-840-2575 x 2206, 2192

TIENES ENTRE 11 Y 20 ANOS…?

PODRIAS PARTICIPAR EN UN ESTUDIO SOBRE EL CICLO MENSTRUAL

Estamos reclutando jóvenes puertorriqueñas para participar en un estudio que nos ayudará a entender las características del ciclo menstrual, la prevalencia de condiciones asociadas y las fuentes de información que usan para educarse sobre este tema.

ESTE ESTUDIO ES ANONIMO. Solo tomará unos 5 minutos contestar las preguntas.

Si tienes preguntas comunícate con la Investigadora Principal del estudio

Dra. Idhaliz Flores Caldera

iflores@psm.edu

Endopr@gmail.com

787-840-2575 x 2206, 2192
